# 

**Published:** 1892-03-26

**Authors:** 


### TO OUR READERS.

ON and from the issue of April 2nd, 1892, THE HOSPITAL will be permanently enlarged, important features will be introduced, and the price raised from **One Penny** to **Twopence**. Those of our readers who like to subscribe direct, by sending their subscriptions to the Publisher, at the Office, 140, Strand, W.C., will be supplied with THE HOSPITAL every week, post free, for 8s. a year; 5s. for half a year; or 2s. 6d. for three months. All subscriptions will be payable in advance.

**Notices of Births, Deaths, and Marriages**, are inserted in this column at a charge of 2s. 6d. for an announcement not exceeding four lines.

### GENERAL CHARITIES.

[This Column is devoted to an account of work of charitable institutions other than Medical Charities. The Editor will be glad to receive reports or news of work at 140, Strand. *Philanthropy* should be plainly written on the envelope.]

The Bishop of Rochester will take the chair at the annual meeting of the Mission to Seamen at Sion College on May 2nd.

Archdeacon Farrar's recent appeal on behalf of the Police Court Missions has been instrumental in bringing a sum of £100 to their coffers.

Mr. W. R. Malcolm has been appointed Treasurer of the National Society for the Prevention of Cruelty to Children, in the room of Mr. Ruthven Pym, retired through ill-health.

Dr. E. Maunde Thompson, Professor R. C. Jebb, M.P., and Mr. Henry Hucks Gibbs, M.P., were elected to serve on the General Committee of the Royal Literary Fund at the recent Annual General Meeting.

The late Mr. James Graham, of Kensington, has bequeathed £3,000 to Dr. Barnardo's Homes and Mission, £2,000 to the National Refuges for Homeless and Destitute Children, and £1,000 to the Pension Fund of the Cabdrivers' Benevolent Association.

The Surgical Aid Society appeals for subscriptions to help on the work in which it is engaged. Though over 101,000 persons have been assisted, much remains to be done to help the numbers of the poor and hardworking population, who cannot afford to purchase proper surgical appliances when in need of them. Donations will be gratefully acknowledged by the Secretary, Salisbury Square, E.C.

The donations in connection with the 109th anniversary dinner of the **BENEVOLENT SOCIETY OF ST. PATRICK** did not reach a very large total. The work done by the Society, which educates at its school in Stamford Street, Blackfriars Road, children of poor Irish parents, irrespective of creed, is one well worthy of support. At the present time some 450 children are receiving such technical training as the resources of the Institution can give.

The financial statement of the **ROYAL GENERAL THEATRICAL FUND** for 1891 is not as satisfactory as the supporters of the Institution would wish for, there being a deficit of £432. It is to be hoped that this will be more than made up for at the annual dinner, under the chairmanship of Mr. George Alexander. The date has not yet been fixed for the annual benefit, which will be given by consent of Sir Augustus Harris at Drury Lane.

Subscriptions to the amount of nearly £900 were received in connection with the celebration of the seventy-third anniversary of the **CITY OF LONDON GENERAL PENSION SOCIETY** which provides permanent relief by means of monthly pensions for artisans, mechanics, manufacturers, tradesmen, above 60 years of age, and their widows above the age of 63. A necessary qualification for the receipt of relief from the society is that applicants must have been employed for at least 20 years in the City of London.

We congratulate the managers of the **SOLDIERS' HOME**, James Street, Buckingham Palace Road, upon the success which has crowned their efforts. It was announced at the Second Anniversary Meeting held last week, that the Home has proved of such benefit to the Guards that steps are already being taken to enlarge it, the Duke of Westminster having offered facilities for the purpose. Nearly £1,200 have already been subscribed for the extension, but more will be gratefully received for this purpose, as well as for the general expenditure of the Home, by the Hon. Treasurer, Sir George Chubb.

It is far from satisfactory that the managers of such an institution as the Field Lane Society should find that the keenest economy fails to keep the expenses within the average annual receipts. The result of this discovery is naturally an appeal to the public for further aid. The institution has a good record of work done during the past forty-nine years, and it is to be hoped its efforts will not be allowed to languish for want of funds. Perhaps modern philanthropists hear a little too much of this constant cry of debt out of which the way at present seems very dark.

A substantial addition was made to the funds of the **BRIXTON ORPHANAGE FOR FATHERLESS GIRLS** at the recent annual meeting over which the Lord Mayor presided. The orphanage, in which children are trained for domestic service, has now entered upon its sixteenth year, during which period 599 children have been enrolled. The inmates of the institution, which is unsectarian in character, are received from all parts of the country, the most necessitous cases being selected for admission as vacancies occur, without having to undergo any canvassing or voting, which is an objectionable feature in the administration of too many charities.

Dr. Barnardo, who was present at the meeting held last week in order to bid farewell to three hundred lads before starting for Canada, drew the attention of the meeting to a fact which cannot be brought to the minds of the public too often. To maintain a criminal lad in gaol is not in itself satisfactory, but it is still less satisfactory that the country should have to pay £8[illegible] a year for such unproductive work. Dr. Barnardo claims, and we believe with justice, that he saves the country much waste by the training and equipping of lads under his system at the very moderate cost of £2[illegible].

### SCRAPS AND GLEANINGS.

THE value of the personal estate of Sir Oscar Clayton has been sworn at £146,745. He leaves an ivory crucifix to the Princess of Wales.

MR. ALEXANDER BRUCE has bequeathed £100 each to the British Orphan Asylum, Earlswood Asylum, and St. John's Foundation School, Leatherhead.

THE Duchess of Albany has expressed her intention of being present at some of the lectures to ladies on Domestic Hygiene, to be given at the Sanitary Institute on and after the 22nd inst.

DURING the past year, 885 children have been treated in the wards of the Victoria Hospital; 196 have been sent to the convalescent branch at Margate, and there have been 44,408 attendances in the out-patient department.

THE London Vegetarian Society will hold a great public debate on Monday, March 28th at eight p.m., in the Library Memorial Hall, Farringdon Street, when they will uphold "Diet and Health Versus Drugs and Disease."

AT the annual meeting of the subscribers to the City of London and East London Dispensary, Wilson Street, Finsbury, it was reported that during the twelve months no fewer than 37,352 poor patients had enjoyed the benefits of the charity.

MR. WILLIAM SALMON, of Penllyne Court, Glamorganshire, who was born in the parish of Wickham Market, Suffolk, last week entered his 103rd year. He is supposed to be the oldest living Freemason, and the oldest member of the Royal College of Surgeons.

IT is, according to present arrangements, probable that whilst the Prince of Wales is the guest of the Earl of Warwick in June next, His Royal Highness will open the new wing at Warnford Hospital, Leamington, which is being erected at a cost of £14,000.

THE Bishop of Chichester presided at the annual meeting of the Alexandra Hospital for Sick Children, Brighton. The report stated that 348 children had been treated as in-patients, and 2,842 as out-patients during the year. The receipts amounted to £1,858 0s. 4d., and the expenditure to £2,089 14s.

TWO HUNDRED AND SEVENTY-TWO POUNDS has been received from old boys of the Boys' Home, Southwark, who are now in Canada, as a thank-offering. In four years £732 has been received from this source. The Institution is preparing to send out 120 lads this spring if sufficient funds are forthcoming.

A FAVOURABLE report was presented at the annual meeting of the Boscombe Hospital. There had been a steady decrease in the expenditure, and an increase in the subscriptions. The building of the new wing is completed at a cost of over £600, affording valuable accommodation to the staff and patients.

THE annual report of the Blackburn and East Lancashire Infirmary for 1891 states 7,496 patients (as against 7,889 in 1890) received treatment. The income for the year was £6,559 5s. 11d., and the expenditure £6,707 11s. Nearly £3,000 has been received towards the Nurses' Home, and building operations will be commenced without delay.

AT the forty-first annual meeting in connection with St. Mary's Hospital, Cambridge-place, Paddington, accounts for the year showed a deficit of £4,346. Prince George of Wales has expressed his willingness to accept the office of president of the hospital, and the foundation stone of the new wing—to be called the "Clarence" wing—will be laid next October.

AT the annual meeting of the Nottingham Samaritan Hospital for Women, the report of the Committee of Management stated the expenditure for the past year had fallen from £834 11s. 2d. to £725 6s. 11d., notwithstanding an increase in the number of patients; 133 in-patients, 2,268 out-patients received treatment, bringing the total since the hospital was opened to 13,623.

BY the will of the late Marchioness of Ailesbury, the trustees of the Savernake Cottage Hospital receive £2,500, of which sum £20 a-year is to be applied towards the maintenance of the Church of England service in the hospital, and the remainder of the income in providing an extra nurse or a lady superintendent with liberty to the trustees to expend £500 in building rooms for the Lady Superintendent.

THE annual report of the Sidcup Cottage Hospital is a gratifying one. The number of cases treated was larger than in any previous year, and the cost per patient less. Eighty-one patients were admitted in the year, and the cost for weekly maintenance was £1 2s. 2d., as against £1 4s. 7d. in 1890, and £1 6s. 4d. in former years. The total of days that patients were in hospital was 2,504 as against 2,061 in 1890.

THE annual report of the Bridgwater Infirmary for the past year is as follows: The number of in-patients admitted was 236, and out-patients 1,612, showing a decrease of 141 on the previous year. Owing to important improvements and other circumstances, the Treasurer is unable to report, as for many years past, a favourable balance in hand, and the year commences with an adverse one of £231 4s. 6d.

AT a meeting of the Executive Committee of the Waifs and Strays' Society, at Church House, Westminster, it was announced that the amount received during the past fortnight was £944 1s. 3d., and the payments for maintenance of homes and boarded-out children to £1,065 7s. 1d. The over-draught on the general fund was £2[illegible]1 5s. 9d. St. Catherine's Home, Southbourne-on-Sea, has been transferred to the Society.

THE 43rd annual report of the Wolverhampton General Hospital shows 2,168 in-patients, and 13,965 out-patients were treated during the past year. The income of the institution was £7,465 19s. 2d., being a decrease of £88 3s. compared with that of 1890. The expenditure amounted to £7,751 6s. 8d., showing an increase of £15 19s. 2d. A new story has been added to the out-patients' department, and an improved service of circulating hot-water pipes carried throughout the building.

THE financial condition of the Aberdeen Lunatic Asylum (including Elmhill House and Daviot Branch) as shown by the annual report of the Board of Directors, is most satisfactory. The income for the past year amounted to £20,520 9s. 5d., and the expenditure to £19,899 3s. 5d. The number of patients under treatment in the asylum was 840, of whom 149 were discharged, 88 recovered, and 26 relieved. The reconstruction of the main building, dating from 1799, is under consideration.

### THE STUDENT OF MEDICINE.

[All communications for this Supplement should be addressed to THE EDITOR of THE HOSPITAL, at 140, Strand, with the words, "Students' Column," written in the left hand top corner of the envelope.]

### APPOINTMENTS.

Eustace Baines, M.B., C.M.Durh., appointed Second Assistant Medical Officer to the St. Marylebone Infirmary. H. M. Bowman, M.D.Lond., M.R.C.S., appointed Senior House-Physician to the National Hospital for the Paralysed and Epileptic (Albany Memorial), Queen Square, Bloomsbury. R. H. Collins, M.R.C.S.Eng., L.R.C.P.Lond., appointed Assistant House-Surgeon to the Poplar Hospital. A. A. Fennings, M.B., B.S., M.R.C.S., L.R.C.P., appointed Junior Medical Officer to the Camberwell House Asylum. C. Gibson, M.D., L.R.C.P., appointed Physician to the Royal Bath Hospital and Rawson Convalescent Home, Harrogate. S. Hillier, M.B., C.M.Edin., appointed First Assistant Medical Officer to the Paddington Infirmary, Harrow Road. A. Hunter, M.B., C.M.Aberd., appointed Junior Assistant Medical Officer to the Montrose Royal Asylum. H. B. Kitchin, M.B., L.R.C.P.Lond., M.R.C.S.Eng., appointed House-Surgeon to the Newport and County Infirmary. J. Liddell, M.D., appointed Physician to the Royal Bath Hospital and Rawson Convalescent Home, Harrogate. A. C. F. Rabagliati, M.D., F.R.C.S.Edin., appointed Honorary Gynæcologist to the Bradford Infirmary. J. W. Smith, L.R.C.P.Lond., M.R.C.S., appointed Honorary Consulting Surgeon to the Doncaster Infirmary. W. S. Tebb, M.D.Camb., L.R.C.P.Lond., M.R.C.S., appointed Honorary Surgeon to the Boscombe Hospital and Provident Dispensary. G. M. Wood, M.B., M.R.C.S., L.R.C.P., appointed Junior House-Physician to the National Hospital for the Paralysed and Epileptic (Albany Memorial), Queen Square, Bloomsbury.

### VACANCIES.

### Resident Medical Officers.

The following vacancies are announced this week, the amount of salary in each case being given after the name of the institution :

Bedford General Infirmary, Surgeon, doubly qualified—Salary £100, Board, &c.

Belgrave Hospital for Children, 79, Gloucester Street, S.W., House-Surgeon—Board, lodging, &c.

Buckingham General Infirmary, Aylesbury, Surgeon and Apothecary, doubly qualified—Salary £80 per annum, rising £10 yearly to £100, Board, &c.

Cardiff Infirmary, Assistant House-Surgeon. Board, &c.

Derbyshire Royal Infirmary, Assistant House-Surgeon. Appointment for six month, but eligible for an additional six months—Salary £10 for first six months, £25 for second six months, Board, &c.

Manchester Southern and Maternity Hospital, House Surgeon.

North Riding Asylum, Clifton, York, Second Assistant Medical Officer.—Salary £100, Board, &c.

Royal Pimlico Dispensary, 104, Buckingham Palace Road, S.W., Medical Officer and Assistant Secretary—Salary £100, with percentage of payments of members, &c.

Salford Union, Assistant Medical Officer for the Union Infirmary, Hope, near Eccles, doubly qualified.—Salary £130, &c.

Township of Manchester, Assistant Medical Officer to the Workhouse at Crumpsall, doubly qualified, unmarried—Salary £100, &c.

### Non-Resident Medical Officers.

London Hospital, Whitechapel Road, E., Surgical Registrar.—Salary £100.

London Lock Hospital and Asylum, Harrow Road, W., and 91, Dean Street, Soho, W., Registrar.

Royal College of Surgeons of England, Member of the Court of Examiners.

### NOTES FROM THE SCHOOLS.

### University of Cambridge.

EXAMINATION IN SANITARY SCIENCE.—Part I. will begin on Tuesday, April 5th, at nine a.m., and will be continued on Wednesday, April 6th, at the same hour. Part II. will be held on Thursday, April 7th, and Friday, April 8th, beginning at nine a.m.; and the practical and oral parts of both examinations will be concluded on Saturday, April 9th.

DEGREES.—At the congregation on March 10th, the following medical degrees were conferred: M.D.—G. C. Fitzgerald, B.A., M.B., Caius; A. C. Ingle, B.A., M.B., B C. M.B. and B.C.—H. E. Durham, B.A., King's; J. G. Hulbert, B.A., Trinity; F. M. Turner, B.A., Trinity; E. H. E. Stack, B.A., Pembroke; M. Craig, B.A., Caius; T. W. Scott, B.A., Caius; J. B. Gillam, B.A., Downing; S. H. Lee, B.A., Downing.

### ATHLETICS.

### Football.

ASSOCIATION CHALLENGE CUP.

ST. MARY'S v. ST. BARTHOLOMEW'S HOSPITAL.—This tie was re-played on March 16th, and after a hard fought game again resulted in a draw of one goal each. The tie will be re-played on Wednesday, March 23rd.

